# Freeze-Thaw Durability of Air-Entrained Concrete

**DOI:** 10.1155/2013/650791

**Published:** 2013-03-21

**Authors:** Huai-Shuai Shang, Ting-Hua Yi

**Affiliations:** ^1^School of Civil Engineering, Qingdao Technological University, Qingdao 266033, China; ^2^State Key Laboratory of Structural Analysis for Industrial Equipment, Dalian University of Technology, Dalian 116024, China; ^3^School of Civil Engineering, Dalian University of Technology, Dalian 116024, China

## Abstract

One of the most damaging actions affecting concrete is the abrupt temperature change (freeze-thaw cycles). The types of deterioration of concrete structures by cyclic freeze-thaw can be largely classified into surface scaling (characterized by the weight loss) and internal crack growth (characterized by the loss of dynamic modulus of elasticity). The present study explored the durability of concrete made with air-entraining agent subjected to 0, 100, 200, 300, and 400 cycles of freeze-thaw. The experimental study of C20, C25, C30, C40, and C50 air-entrained concrete specimens was completed according to “the test method of long-term and durability on ordinary concrete” GB/T 50082-2009. The dynamic modulus of elasticity and weight loss of specimens were measured after different cycles of freeze-thaw. The influence of freeze-thaw cycles on the relative dynamic modulus of elasticity and weight loss was analyzed. The findings showed that the dynamic modulus of elasticity and weight decreased as the freeze-thaw cycles were repeated. They revealed that the C30, C40, and C50 air-entrained concrete was still durable after 300 cycles of freeze-thaw according to the experimental results.

## 1. Introduction 

Concrete is considered as one of the most nonhomogeneous and demanding engineering materials used by mankind. The durability [1–5] of concrete is defined as the ability to withstand damaging effects of environment without deterioration for a certain period of time. The durability of concrete involves resistance to frost, corrosion, permeation, carbonation, stress corrosion, chemical attack, and so on. 

Concrete has a potential to be damaged if it is subjected to freeze-thaw cycles. The American Concrete Institute (ACI) has established specifications for protection of concrete placed during cold weather. ACI defined cold weather as the period where more than three successive days have a mean daily air temperature less than 40 F (Fahrenheit). The freeze-thaw durability of concrete is of utmost importance in countries having subzero temperature conditions, such as The Arctic Zone, Russia, Northern China, and China. Frost damage, a progressive deterioration which starts from the surface separation or scaling and ends up with complete collapse, is a major concern when concrete is used in colder regions. The deterioration proceeds as freezing and thawing cycles are repeated, and the material gradually loses its stiffness and strength. In addition, the increasing irreversible expansion is induced. So frost damage is a very complex fatigue process. It has been a significant scientific and technical problem to improve the freeze-thaw durability and to prolong the service life of concrete. 

Hong-Qiang et al. [4] and Li-kun [5] investigated the relative dynamic modulus of elasticity (RDME) and weight loss of plain concrete after different cycles of freeze-thaw. Sun et al. [6] investigated the loss of dynamic elastic modulus of high-strength concrete under the action of load and freeze-thaw cycles. Zaharieva et al. [7] investigated the influence of freeze-thaw cycles on the loss of dynamic elastic modulus of recycled aggregate concrete. The effect of sodium chloride solution, freeze-thaw cycling, and externally applied load on the relative dynamic modulus of elasticity (RDME) and weight loss of concrete was experimentally investigated by Sun et al. [8]. Cohen et al. [9] investigated the relative dynamic modulus of elasticity (RDME) and weight loss of non-air-entrained high-strength concrete after freeze-thaw cycles. 

 Air-entraining agent [10–13] was recommended for nearly all concretes, principally to improve resistance to freeze-thaw cycles when exposed to water and deicing chemicals in cold regions. Very little work has been documented on the freezing and thawing durability of air-entrained concrete. This paper presents experimental study on the relative dynamic modulus of elasticity and weight loss of C20, C25, C30, C40, and C50 air-entrained concretes after 0, 100, 200, 300, and 400 cycles of freeze-thaw according to “the test method of long-term and durability on ordinary concrete” GB/T50082-2009 [14]. And the influence of freeze-thaw cycles on the relative dynamic modulus of elasticity and weight loss of C20, C25, C30, C40, and C50 air-entrained concrete was analyzed according to the experimental results. 

## 2. Experimental Procedures

### 2.1. Materials and Mix Proportions

In this investigation, local materials were utilized. A Chinese standard (GB175-99) [15] Portland cement 425 (which has standard compressive strength of 42.5 MPa at the age of 28 days) was used. Natural river sand with fineness modulus of 2.6 was used. Coarse aggregate was a crushed stone with diameter from 5 mm~20 mm. The mix proportions are listed in Table 1. The mixing started after putting all the coarse aggregate and fine aggregate into the mixer. These ingredients were mixed for about 1 min, and then the water with air-entraining agent was added in 1 minute. Finally the mixing continued for about 2 min after all water was added.

### 2.2. Test Specimens and Testing Programs

 Concrete prisms with size of 100 mm × 100 mm × 400 mm (to determine the weight loss and the dynamic modulus of elasticity) were prepared. The specimens were cast in steel molds and compacted through external vibration and demolded 24 h later. All the specimens were cured in a condition of 20 ± 3°C and 95 percent RH for 23 days. Thereafter, the specimens were immersed in water for 4 days prior to the freeze-thaw cycles. Then when the age of the specimens was 28 days, the air-entrained concrete specimens were placed into the freeze-thaw apparatus.

 In this paper, the freeze-thaw test apparatus [16] meeting the requirement of “the test method of long-term and durability on ordinary concrete” GB/T 50082-2009 was used. The freeze-thaw cycles consisted of alternately lowering the temperature of the specimens from 6°C to −15°C and raising it from −15°C to 6°C, while the temperature of the antifreeze ranged from 8 ± 2°C to −17 ± 2°C and then warms to 8 ± 2°C all within 2.5~3 hours.

The dynamic modulus of elasticity and weight loss of each specimen were measured before placing the specimens with size of 100 mm × 100 mm × 400 mm into the freeze-thaw apparatus. One specimen with size of 100 mm × 100 mm × 400 mm was placed in a rubber container. In which, standard concrete prisms were surrounded by water. Specimens were removed for testing when they were in a thawed condition at 50-cycle or 100-cycle intervals. The dynamic modulus of elasticity and weight were recorded. Before returning the specimens into the freeze-thaw apparatus in a random order, containers were cleaned out and fresh water was added. The C20 and C25 air-entrained concrete specimens were exposed to 300 cycles of freeze-thaw, the C30, C40, and C50 air-entrained concrete specimens were exposed to 400 cycles of freeze-thaw.

## 3. Results and Discussions

**Figure 1:**
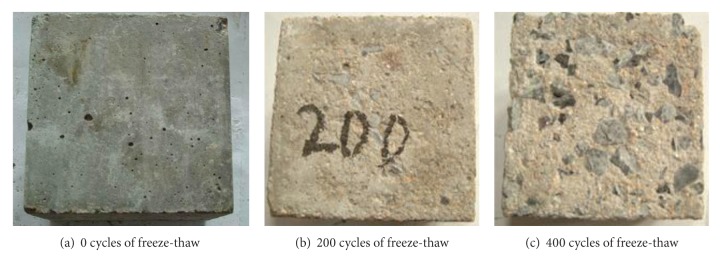
Surface of C30 air-entrained concrete after 0, 200, and 400 cycles of freeze-thaw.

The surface deterioration of the C30 air-entrained concrete specimens undergoing 0, 200, and 400 cycles of freeze-thaw is shown in Figures 1(a), 1(b), and 1(c). The microcracks were caused after the action of freezing and thawing cycles, and then the coarse aggregates and cement part were separated because of the action of freeze-thaw cycles. So the surface separation or scale off was caused by freeze-thaw cycles just as shown in Figures 1(b) and 1(c). 

### 3.1. The Relative Dynamic Modulus of Elasticity

The RDME of C20, C25, C30, C40, and C50 air-entrained concrete after different cycles of freeze-thaw was given in Table 2.

The relative dynamic modulus of elasticity is defined as follows:

(1) $$\begin{array}{c} P=\frac{f_{n}}{f_{0}}\times 100, \end{array}$$

where *P* is relative dynamic modulus of elasticity at *n* cycles of freeze-thaw, expressed as percentage, computed as the average of three specimens; *f*_*n*_ is dynamic modulus of elasticity at *n* cycles of freeze-thaw; *f*_0_ is dynamic modulus of elasticity before freeze-thaw cycles.

**Figure 2:**
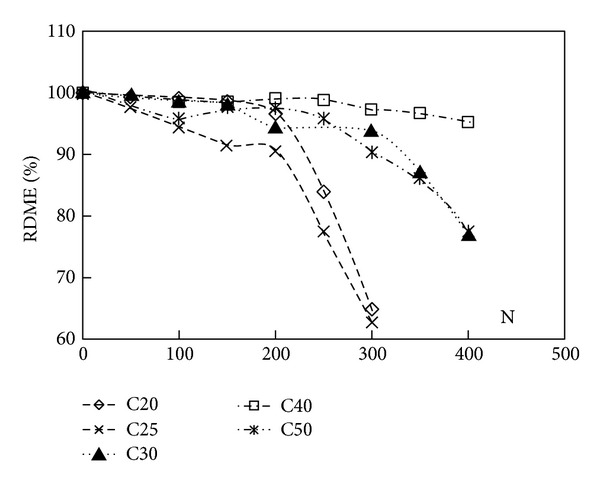
RDME of air-entrained concrete after different cycles of freeze-thaw (%).

After 300 cycles of freeze-thaw, the C30, C40, and C50 air-entrained concrete specimens showed a small loss of RDME, while C20 and C25 air-entrained concrete specimens showed considerable loss of RDME, as shown in Figure 2.

As seen from Table 2 and Figure 2, for C20, C25, C30, C40, and C50 air-entrained concrete, the RDME decreased slowly during the first 200 freeze-thaw cycles; the RDME of C20, C25, C30, C40, and C50 air-entrained concrete were 96.70, 90.75, 94.60, 99.05, and 97.50 percent after 200 cycles of freeze-thaw. In subsequent cycles of freeze-thaw, it is observed that the deterioration proceeds quickly. And after 300 freeze-thaw cycles, the RDME of C20 and C25 air-entrained concrete gave an obvious decrease; it decreased to about 64.95 and 62.80 percent, while the RDME of C30 and C40 air-entrained concrete only gave a decrease of 6.10, 2.65, and 9.65 percent. From 300 to 400 cycles of freeze-thaw, the RDME of C30 and C50 air-entrained concrete gave a decrease of 16.85 and 12.75 percent. The good durability of C30, C40, and C50 air-entrained concrete compared to the C20, C25 air-entrained concrete can be attributed to its higher compressive strength.

Sun et al. [8] and Zaharieva et al. [7] investigated the influence of freeze-thaw cycles on the RDME of plain concrete. The conclusion that the RDME decreased to 62 percent after 100 cycles of freeze-thaw was given by Li-kun. Hong-Qiang et al. found that the RDME decreased to 64 percent after 100 cycles of freeze-thaw. The dynamic modulus of elasticity is the proportion of stress to strain when the stress is least under dynamic loads. It can be measured by means of longitudinal vibration or flexural vibration. It reflects the elasticity performance of material, similarly to the initial tangential modulus under static loads. The loss of the dynamic modulus of elasticity with freeze-thaw cycles means the loss of the elasticity performance. Therefore, the influence of the freeze-thaw cycles on the RDME of plain concrete is higher than that on the RDME of the air-entrained concrete. 

### 3.2. Weight Loss

One type of deterioration of concrete structures by cyclic freeze-thaw is surface scaling. Surface scaling is the loss of paste and mortar from the surface of concrete by the cyclic freeze-thaw or by an internal reaction of aggregate (e.g. alkali-silica reaction in concrete mixed with alkali-reactive aggregate). In extreme cases, the loss of paste can result in loosening of coarse aggregate and gradual reduction in strength of concrete structures. The weight loss will be caused by surface scaling, so the weight loss was measured. 

**Figure 3:**
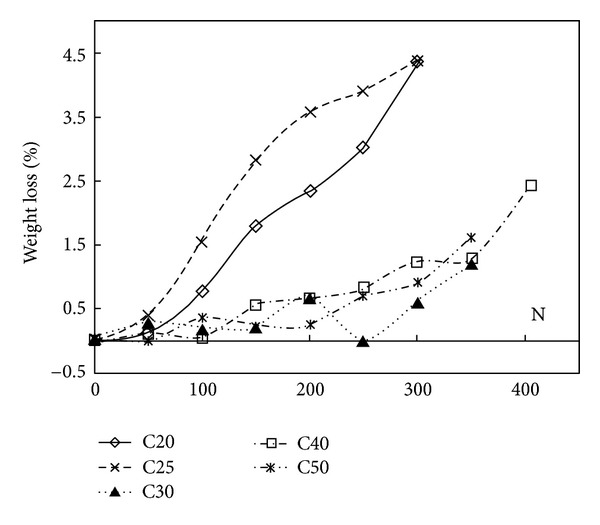
Effect of freeze-thaw cycles on weight loss of air-entrained concrete.

Table 3 gives the weight of air-entrained concrete after different cycles of freeze-thaw. The weight loss for air-entrained concrete versus the number of freeze-thaw cycles is shown in Figure 3. The weight loss is defined as follows:

(2) $$\begin{array}{c} \Delta W_{n}=\frac{G_{0}-G_{n}}{G_{0}}\times 100, \end{array}$$

where Δ*W*_*n*_ is weight loss at *n* cycles of freeze-thaw, expressed as percentage, computed as the average of three specimens; *G*_*n*_ is weight at *n* cycles of freeze-thaw; *G*_0_ is weight before freeze-thaw cycles.

It can be seen from Table 3 and Figure 3 that the influence of freeze-thaw cycles on the weight loss of C20 and C25 air-entrained concrete is larger than that on the weight loss of C30, C40 and C50 air-entrained concrete. After 200 cycles of freeze-thaw, the weight loss was 0.70, 0.67, and 0.25 percent for C30, C40, and C50 air-entrained concrete, while the weight loss was 2.35 and 3.58 percent for C20 and C25 air-entrained concrete. 

The test results show that the weight loss of C30 air-entrained concrete was 0.20 percent compared with 1.81 percent for plain concrete after 100 cycles of freeze-thaw [4]. For C20, C25, C30, C40, and C50 air-entrained concrete, the maximum of weight loss was only 4.38 percent after 300 cycles of freeze-thaw. While Hong-Qiang et al. [4] found that the weight loss of C30 plain concrete decreased to 3.74 percent after 125 cycles of freeze-thaw. For plain concrete, the weight increase was observed by Hong-Qiang [4] and Li-kun [5] during the first 25 freeze-thaw cycles. For air-entrained concrete, the weight increase wasnot observed. 

The weight loss of concrete specimens is caused by surface separation or scale off. The weight variation during freeze-thaw cycles is due to movement in and out of water in the specimen and surface separation or scaling (surface scaling is the loss of paste and mortar from the surface of concrete by the cyclic freeze-thaw). As soon as microcracking takes place, the deteriorated zones filled with the surrounding water will cause change in the weight of the specimen. If the mass of surface separation is larger than the water absorbed by the concrete specimens, the weight of the concrete specimens will increase. The weight of the concrete specimens will decrease when the mass of surface separation is less than the water absorbed by the concrete specimens. Compared with plain concrete, the deteriorated zones filled with the surrounding water occurred in the air-entrained concrete needed much more cycles of freeze-thaw.

In actual concrete structures, concrete surface scaled markedly when exposed to deicing salt and freeze-thaw cycles caused by the change of climate. The cycling rate in the laboratory conditions was much higher than that in the natural environment because of the fast change of temperature. Thus, it is reasonable that the scaling observed during the tests was more severe, and the scaling depth of concrete specimens was over 1 mm. 

### 3.3. The Ultrasonic Velocity

A lot of structures, like bridges, tunnels, dams, buildings, and others, were constructed with concrete material. During the life cycle of these structures, degradations can occur because of mechanical, thermal, or chemical stresses. These often lead to the development of porosity, microcracks, and cracks in the material. Knowing the concrete structure state to prevent or repair damage is needed so the nondestructive characterisation is an important way, and the ultrasonic method is often proposed.

In this work, the ultrasonic velocity of C30 air-entrained concrete was measured with ultrasonic method according to “Testing Code of Concrete for Port and Waterwog Engineering” JTJ 270-98 [17].  Table 4 gives the decreasing percentage of the ultrasonic velocity of air-entrained concrete after different cycles of freeze-thaw. It can be seen from Table 4 that the ultrasonic velocity decreased slowly during the first 200 freeze-thaw cycles, and it gave only about a 2.4 percent decrease over the initial value. However, in subsequent freeze-thaw cycles, it is observed that the deterioration usually proceeds. And after 400 freeze-thaw cycles, it decreased to about 84.7 percent of the initial value. 

### 3.4. Discussion

Concrete is a three-phase composite structure at microscopic scale, a cement matrix, aggregate, and the interfacial transition zone between the two. The microcracks will be caused by the action of freezing and thawing cycles; the direction and distribution of microcosmic cracks are stochastic. The microcosmic cracks manifold and become broad as freeze-thaw cycles are repeated. Air-entrained concrete contains billions of microscopic air cells when air-entraining agents were used in concrete. These relieve internal pressure on the concrete by providing tiny chambers for the expansion of water when it freezes. So, comparing the test results in this paper with the conclusion given by other authors [4, 5], the deceased percentage for the relative dynamic modulus of elasticity and weight loss of air-entrained concrete is less than that of plain concrete after the same cycles of freeze-thaw. It means that the deterioration of freeze-thaw durability for air-entrained concrete is slower than that of plain concrete. It is because the mixed air-entraining agent in concrete can make them up effectively and thus improve the freeze-thaw durability.

## 4. Conclusion

The effects of freeze-thaw cycles on the RDME and weight loss of C20, C25, C30, C40, and C50 air-entrained concrete were investigated. Based on the experimental work in this study and the discussion about the experimental results, the results of the investigation can be summarized as follows

- The RDME decreased as the freeze-thaw cycles were repeated. After 100 cycles of freeze-thaw, the RDME decreased to 94.35 and 98.75 percent for C25 and C30 air-entrained concrete, and 64 percent for C30 plain concrete. Therefore, the freeze-thaw durability of air-entrained concrete is much higher than that of plain concrete.
- After 200 cycles of freeze-thaw, the weight loss was 0.70, 0.67, and 0.25 percent for C30, C40, and C50 air-entrained concrete, and 2.35 and 3.58 percent for C20 and C25 air-entrained concrete. The weight variation during freeze-thaw cycles is due to moveming in and out of water in the specimen and surface separation or scaling.
- The freeze-thaw durability of plain concrete is poor, but it can be improved greatly when air-entraining agent is mixed into concrete. It demonstrates that ordinary strength concrete can also have a high freeze-thaw durability.

## Figures and Tables

**Table 1:** The mix proportion of air-entrained concrete in per cubic meter.

	Cement (MPa)	W/C	Cement (kg/m^3^)	Sand (kg/m^3^)	Coarse aggregate (kg/m^3^)	Water (kg/m^3^)	Air-entraining agent (kg/m^3^)	Air content (%)
C20	32.5	0.40	339.00	642.00	1185.20	133.80	0.85	5.5~6.5
C25	32.5	0.40	356.00	615.20	1188.00	141.00	0.89	5.5~6.5
C30	42.5	0.40	412.67	586.83	1186.00	164.30	1.03	5.5~6.5
C40	42.5	0.36	467.60	568.20	1148.00	166.00	1.17	5.5~6.5
C50	42.5	0.32	526.00	520.00	1154.80	168.30	1.30	5.5~6.5

**Table 2:** RDME of air-entrained concrete after different cycles of freeze-thaw (%).

	Number of freeze-thaw cycles								
	0	50	100	150	200	250	300	350	400
C20	100	99.45	99.4	98.75	96.7	83.85	64.95	/	/
C25	100	97.60	94.35	91.55	90.75	77.35	62.8	/	/
C30	100	99.55	98.75	98.2	94.6	/	93.9	87.3	77.05
C40	100	/	98.4	98.55	99.05	98.9	97.35	96.75	95.4
C50	100	/	95.85	97.6	97.5	95.8	90.35	85.95	77.6

“/” means: “the measurements were not made.”

**Table 3:** Weight of air-entrained concrete after different cycles of freeze-thaw (Kg).

	Number of freeze-thaw cycles								
	0	50	100	150	200	250	300	350	400
C20	8.930	8.920	8.860	8.770	8.720	8.660	8.540	/	/
C25	9.417	9.380	9.270	9.150	9.080	9.050	9.005	/	/
C30	9.960	9.930	9.940	9.940	9.890	/	9.900	9.840	9.685
C40	9.740	9.730	9.735	9.685	9.675	9.660	9.410	9.615	9.510
C50	9.960	/	9.925	9.940	9.935	9.890	9.870	9.800	9.585

“/” means: “the measurements were not made.”

**Table 4:** Decreasing percentage of the ultrasonic velocity after freeze-thaw cycles.

Number of freeze-thaw cycles	0	100	200	300	400
Loss of the ultrasonic velocity (%)	100	97.7	97.6	91.0	84.7
